# Mobilome of *Brevibacterium aurantiacum* Sheds Light on Its Genetic Diversity and Its Adaptation to Smear-Ripened Cheeses

**DOI:** 10.3389/fmicb.2019.01270

**Published:** 2019-06-10

**Authors:** Sébastien Levesque, Alessandra G. de Melo, Simon J. Labrie, Sylvain Moineau

**Affiliations:** ^1^Département de Biochimie, de microbiologie, et de Bio-informatique, Faculté des Sciences et de Génie, Groupe de Recherche en Écologie Buccale, Faculté de Médecine Dentaire, Université Laval, Quebec City, QC, Canada; ^2^Syntbiolab, Lévis, QC, Canada; ^3^Centre de Référence pour Virus Bactériens Félix d’Hérelle, Faculté de Médecine Dentaire, Université Laval, Quebec City, QC, Canada

**Keywords:** *Brevibacterium aurantiacum*, smear-ripened cheeses, comparative genomics, mobile genetic elements, iron acquisition

## Abstract

*Brevibacterium aurantiacum* is an actinobacterium that confers key organoleptic properties to washed-rind cheeses during the ripening process. Although this industrially relevant species has been gaining an increasing attention in the past years, its genome plasticity is still understudied due to the unavailability of complete genomic sequences. To add insights on the mobilome of this group, we sequenced the complete genomes of five dairy *Brevibacterium* strains and one non-dairy strain using PacBio RSII. We performed phylogenetic and pan-genome analyses, including comparisons with other publicly available *Brevibacterium* genomic sequences. Our phylogenetic analysis revealed that these five dairy strains, previously identified as *Brevibacterium linens*, belong instead to the *B. aurantiacum* species. A high number of transposases and integrases were observed in the *Brevibacterium* spp. strains. In addition, we identified 14 and 12 new insertion sequences (IS) in *B. aurantiacum* and *B. linens* genomes, respectively. Several stretches of homologous DNA sequences were also found between *B. aurantiacum* and other cheese rind actinobacteria, suggesting horizontal gene transfer (HGT). A HGT region from an iRon Uptake/Siderophore Transport Island (RUSTI) and an iron uptake composite transposon were found in five *B. aurantiacum* genomes. These findings suggest that low iron availability in milk is a driving force in the adaptation of this bacterial species to this niche. Moreover, the exchange of iron uptake systems suggests cooperative evolution between cheese rind actinobacteria. We also demonstrated that the integrative and conjugative element BreLI (*Brevibacterium* Lanthipeptide Island) can excise from *B. aurantiacum* SMQ-1417 chromosome. Our comparative genomic analysis suggests that mobile genetic elements played an important role into the adaptation of *B. aurantiacum* to cheese ecosystems.

## Introduction

The coexistence and succession of diverse microorganisms over time lead to the development of distinct organoleptic properties in a wide variety of aged cheeses (Irlinger and Mounier, 2009). During the ripening period, the surface of smear-ripened cheeses is regularly washed with a microbial-rich brine, which allows the colonization of an orange microbial mat composed of various species of yeasts and bacteria (Brennan et al., 2002). The early surface microbiota of washed-rind cheeses is primarily composed of yeasts that release growth factors and alkalinize the cheese surface by metabolizing the lactate produced by lactic acid bacteria added as starter cultures (Alper et al., 2013). These processes promote the growth of acid-sensitive, salt-tolerant bacterial strains primarily composed of coryneform actinobacteria from the *Micrococcaceae, Corynebacteriaceae*, and *Brevibacteriaceae* families (Brennan et al., 2002). The combined metabolic activities of this complex microbiota are responsible for the typical texture, color, and aroma of this type of cheeses (Schröder et al., 2011).

Strains of *Brevibacterium linens* have been acknowledged as widely used for washed rind cheese ripening as they produce volatile sulfur compounds (VSCs) (Amarita et al., 2004; Hanniffy et al., 2009), carotenoids (Krubasik and Sandmann, 2000; Leclercq-Perlat et al., 2004), lipolytic and proteolytic enzymes (Rattray and Fox, 1999; Leclercq-Perlat et al., 2000), which drive the development of the organoleptic features of smear-ripened cheeses. The dairy strains previously identified as *B. linens* ATCC 9175 and *B. linens* BL2 were reported to produce VSCs and carotenoids (Amarita et al., 2004; Dufossé and De Echanove, 2005; Hanniffy et al., 2009) but they were separated from the type strain *B. linens* ATCC 9172 and reclassified as *Brevibacterium aurantiacum* according to their genetic, physiological, and biochemical characteristics (Gavrish et al., 2004; Forquin et al., 2009). A recent study also identified *B. aurantiacum* as the dominant bacterial species in five different smear-ripened cheeses (Cogan et al., 2014), suggesting that the taxonomic position of industrial *Brevibacterium* cheese isolates may need to be revisited.

Traditionally, the cheese ripening process involved the transfer of an undefined microbiota from mature to fresh unripened cheeses, thereby selecting for microorganisms well adapted to this ecosystem (Monnet et al., 2010). Hence, smear-ripened cheese is an interesting model to study the adaptation of microorganisms to a new habitat. The prevalence of horizontal gene transfer (HGT) in cheese rind microbiomes has recently been described, with genes involved in iron siderophore acquisition shown to be widespread in the rind microbiomes of cheeses from Europe and the United States (Bonham et al., 2017). Interestingly, the low iron concentration found in cheese rinds appears to be one of the major metabolic determinants for the growth of ripening bacteria (Noordman et al., 2006; Monnet et al., 2012). In addition, a recent study performing the comparative analysis of 23 *Brevibacterium* spp. genomes, including 12 isolates from cheeses, identified genetic determinants involved in adaptation to the cheese habitat (Pham et al., 2017). The gene repertoire of *Brevibacterium* spp. involved in iron acquisition, osmotic tolerance, and bacteriocin production has been described as well as the ability of this microbial group to use the energy compounds present in cheeses, such as carbohydrates, amino acids, proteins, and lipids (Pham et al., 2017).

While our knowledge on the factors involved on *Brevibacterium* spp. niche adaptation has broaden, we still have a limited comprehension of the mobilome of these species. In fact, most *Brevibacterium* spp. isolated from cheeses previously studied were sequenced using short-read sequencing and their genome sequences remained at the draft level. As such, an in-depth investigation of the mobile genetic elements could not be performed as the analysis of transposon-rich genomes, for example, critically depend on long read sequencing technology (Koren et al., 2013; Pfeiffer et al., 2018).

Here, we report the complete genome sequences of five industrial dairy strains of *B. aurantiacum* and one strain of *B. linens* using the PacBio RSII platform. We performed 16S rRNA and core-genome phylogenetic analyses, which led to the reclassification of dairy strains into the *B. aurantiacum* species. Additionally, we carried out comparative analysis to describe the mobilome of *B. aurantiacum* involved in genome plasticity and adaptation of this species to smear-ripened cheeses.

## Materials and Methods

### Bacterial Strains and DNA Sequencing

*Brevibacterium* strains used in this study are listed in Table 1. They were routinely cultivated in Luria Bertani (LB) or Brain Heart Infusion (BHI) medium at 30°C with agitation (200 rpm). Pulsed-Field Gel Electrophoresis (PFGE) was performed as described previously (Le Bourgeois et al., 1989; Simpson et al., 2003), using the restriction enzymes AseI and PsiI. Genomic DNA was purified using a QIAGEN Genomic tip 20/G kit, according to manufacturer instructions. Single Molecule Real-Time (SMRT) sequencing was performed on a PacBio RSII sequencer (Génome Québec Innovation Centre). Basic Local Alignment with Successive Refinement (BLASR) (Chaisson and Tesler, 2012) was used to align and preassemble the sequences using the longest reads as seeds to which the other subreads were recruited and mapped to correct random errors. Celera assembler (Myers et al., 2000) was used to assemble long and corrected reads into contigs. The sequences were refined using Quiver and genomes were then assembled into one contig using the Hierarchical Genome Assembly Process (HGAP) (Chin et al., 2013). The native plasmid pBLA8 from *B. linens* ATCC 19391 was purified using a QIAGEN Plasmid Maxi kit according to the manufacturer’s instructions. Genome libraries were also made using the Nextera XT DNA library preparation kit (Illumina) and sequenced using a MiSeq reagent kit v2 (Illumina, 500 cycles) on a MiSeq system. *De novo* assembly was performed with Ray assembler version 2.2.0 (Boisvert et al., 2010). Nucleotide coverage, ranging from 3,700× to more than 20,000× for each nucleotide, was calculated with SAMtools (Li et al., 2009).

**Table 1:** *Brevibacterium* spp. strains used in this study.

Strain	GenBank numbers	Source	Sequencing	Year	Genome status	References
			technology			
***B. aurantiacum***		
SMQ-1417	CP025330	Dairy strain	PacBio SMRT	2017	Complete	This study
SMQ-1418	CP025331	Dairy strain	PacBio SMRT	2017	Complete	This study
SMQ-1419	CP025333	Dairy strain	PacBio SMRT	2017	Complete	This study
SMQ-1420	CP025334	Dairy strain	PacBio SMRT	2017	Complete	This study
SMQ-1421	CP025332	Dairy strain	PacBio SMRT	2017	Complete	This study
JB5	NZ_NRGX01000001.1	Dairy isolate	PacBio SMRT	2017	Complete	Bonham et al., 2017
SMQ-1335	NZ_CP017150.1	Dairy strain	PacBio SMRT	2016	Complete	Melo et al., 2016
***B. linens***		
ATCC 19391	CP026734	Unknown	PacBio SMRT	2017	Complete	Leret et al., 1998
***B. epidermidis***		
BS258	NZ_CP014869.1	Marine sediment	PacBio SMRT	2016	Complete	Zhu et al., 2016
**Additional *B. aurantiacum* draft genomes used for the pan-genome analysis**						
**Strain**	**WGS accession numbers**	**Source**	**Sequencing**	**Year**	**Genome status**	**References**

			**technology**			
ATCC 9175	FXZB01000001: FXZB01000070	Camembert cheese	Illumina MiSeq	2017	Permanent draft (70 contigs)	Pham et al., 2017
CNRZ 920	FXZG01000001: FXZG01000073	Beaufort cheese	Illumina MiSeq	2017	Permanent draft (73 contigs)	Pham et al., 2017
6 (3)	FXZI01000001: FXZI01000097	Langres cheese	Illumina MiSeq	2017	Permanent draft (97 contigs)	Pham et al., 2017
8 (6)	FXYZ01000001: FXYZ01000091	Reblochon cheese	Illumina MiSeq	2017	Permanent draft (91 contigs)	Pham et al., 2017

### General Genome Features Prediction

The *Ori* regions were set upstream of the gene coding for the chromosomal replication initiator protein DnaA, as previously described (Melo et al., 2016). Gene prediction and annotation were performed with the NCBI prokaryotic genome annotation pipeline (Tatusova et al., 2016; Haft et al., 2018). Gene annotation was also performed separately with the RAST server (Overbeek et al., 2014) and BLASTp (Altschul et al., 1997). Table 2 presents the general genome features of *Brevibacterium* strains. For pBLA8, open reading frames (ORF) were identified with GeneMark.hmm (Besemer et al., 2001) while BLASTp (Altschul et al., 1997) was used to predict ORF function. The presence of CRISPR (Clustered Regularly Interspaced Short Palindromic Repeats) arrays and their associated (*cas*) genes was assessed using CRISPRCasFinder (Couvin et al., 2018). Additionally, genes related to the production of bacteriocins or antimicrobial post-translational modified peptides were predicted using Bagel4 (Van Heel et al., 2018). Prophages prediction was performed using PHASTER (Arndt et al., 2016) coupled with manual inspection of the regions flanking putative prophages, especially when no genes encoding structural proteins were identified.

**Table 2:** General genome features of *B. aurantiacum, B. linens* and *B. epidermidis*.

Strain	Genome length (Mb)	% G+C content	Predicted CDS	Hypothetical protein (%)	Assigned function (%)	rRNA	tRNA
***B. aurantiacum***		
SMQ-1335	4.210	62.63	3 807	21.4	78.6	12	49
SMQ-1417	4.424	62.76	3 990	21.2	78.8	12	49
SMQ-1418	4.193	62.77	3 751	19.7	80.3	12	50
SMQ-1419	4.039	62.71	3 668	21.2	78.8	12	49
SMQ-1420	4.329	62.73	3 874	21.1	78.9	12	49
SMQ-1421	4.085	62.76	3 687	20.1	79.9	12	49
JB5	4.311	62.79	3 859	20.1	79.9	12	49
***B. linens***		
ATCC 19391	3.807	64.79	3 377	23.7	76.3	12	48
***B. epidermidis***		
BS258	3.862	64.16	3 364	19.1	80.9	12	47

### Phylogenetic Analysis of *B. linens* and *B. aurantiacum*

**FIGURE 1:**
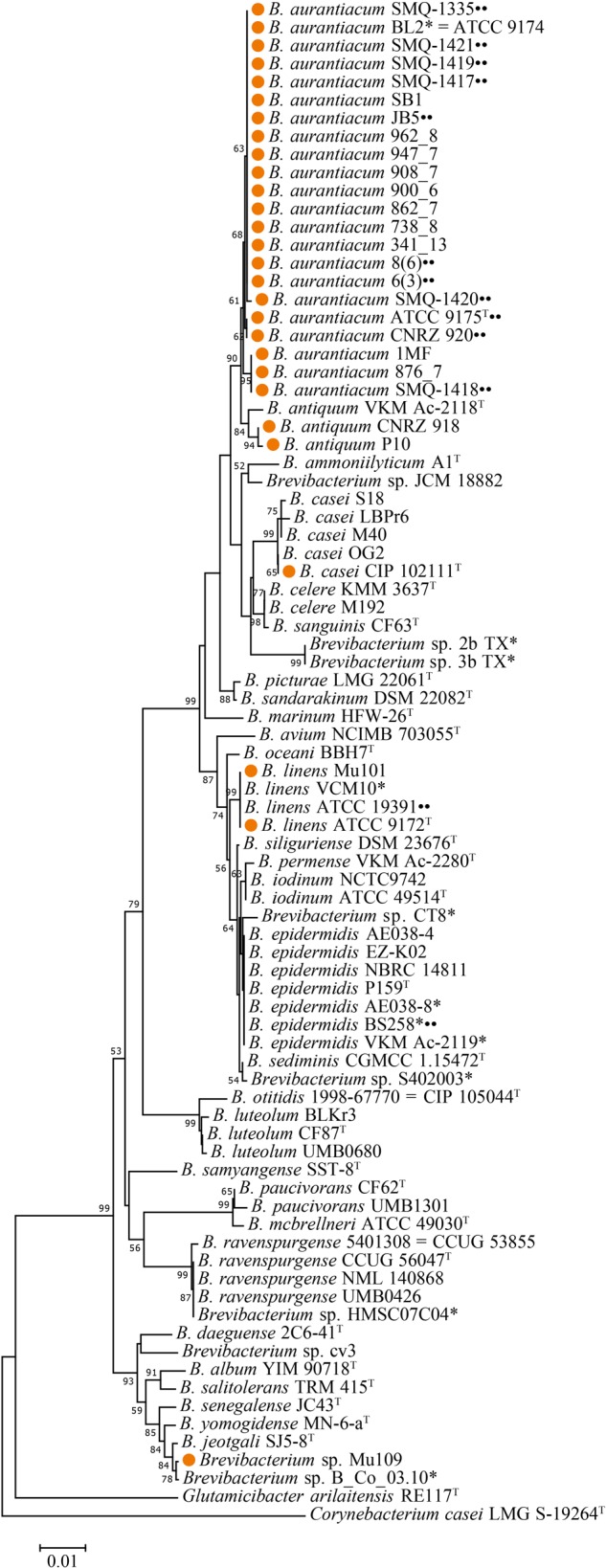
Phylogenetic tree of 82 *Brevibacterium* spp. 16S rRNA nucleotide sequences. The phylogenetic tree was constructed with MEGA 7.0.26 using the neighbor joining method with 1,000 bootstraps (only bootstrap values >50 are shown next to the nodes). *Brevibacterium* dairy strains have their names preceded by orange circles. Strains used in this study are marked with two black circles. Type strains are identified with T superscript. Strains marked with an asterisk should have the taxonomic classification revisited on GenBank. *C. casei* LMG S-19264 and *Glutamicibacter arilaitensis* RE117 were used as out-groups.

We used a broad phylogenetic distribution of 82 *Brevibacterium* spp. 16S rRNA gene sequences from the NCBI database, including the strains described in this study, to perform a phylogenetic analysis using MEGA 7.0.26 software (Kumar et al., 2016). *Glutamicibacter arilaitensis* RE117 and *Corynebacterium casei* LMG S-19264 were used as out-groups. We trimmed the sequences to start and finish in the same conserved regions using Geneious^®^ 11.1.2 (Biomatters, New Zealand). We then aligned the sequences with Multiple Sequence Comparison by Log-Expectation (MUSCLE) (Edgar, 2004). The phylogenetic tree (Figure 1) was constructed using the neighbor-joining method (Saitou and Nei, 1987; Tamura et al., 2004). We performed the analysis with 1,000 bootstraps and the percentage of trees in which the associated taxa clustered together is shown next to the branches (Felsenstein, 1985). The tree is drawn to scale with branch lengths measured in the number of substitutions per site. All positions containing gaps and missing data were eliminated and there were a total of 1,165 positions in the final dataset.

### Core and Pan-Genome Analyses

**FIGURE 2:**
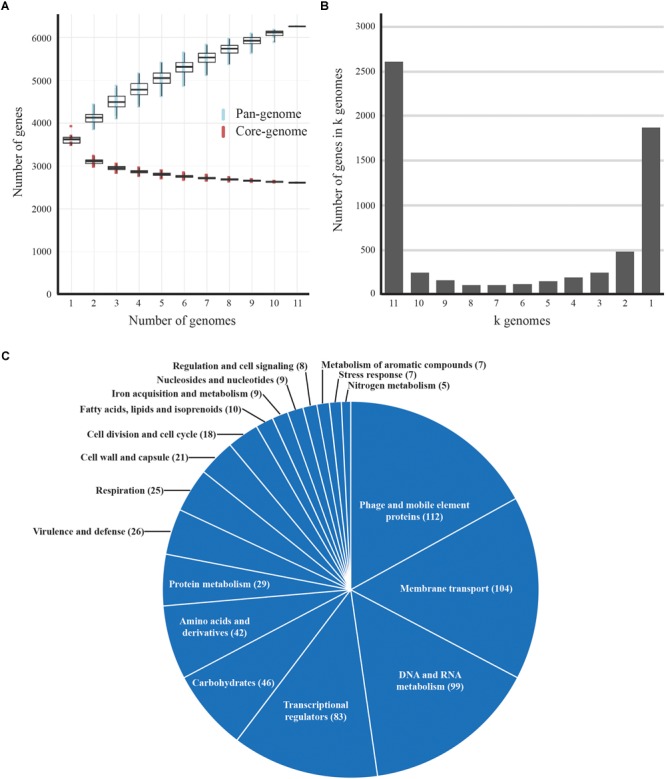
Pan-genome analysis of *B. aurantiacum*. **(A)** Accumulated number of new genes in the pan-genome and genes attributed to the core-genome are plotted against the number of added genomes. **(B)** Accumulated number of genes in *k* genomes are plotted against the number of *k* genomes. The number of core genes present in all 11 *B. aurantiacum* genomes can be observed with *k* = 11 genomes. **(C)** Functional classification of *B. aurantiacum* orphan genes in RAST subsystem categories. Similar metabolic subsystem categories are fused together and only categories with >5 gene counts are shown. Proteins with hypothetical and putative function are not shown.

The protein sequences of seven complete and four draft genomes of *B. aurantiacum* were extracted from GenBank and added to the pan-genome analyses. All genomes used in our analyses were annotated with the NCBI pipeline. The clustering of the protein sequences in orthologous groups was conducted with USEARCH (Edgar, 2010), requiring greater than 60% identity with alignment over more than 75% of the protein sequence. In-house Python scripts were used to extract the pan- and the core-genome of the *B. aurantiacum* strains. Figure 2 illustrates the core- and pan-genome generated with in-house R scripts using ggPlot2 (Ito and Murphy, 2013). Genes present in only one genome (ORFans) were classified into Rapid Annotation using Subsystem Technology (RAST) subsystem categories (Overbeek et al., 2014; Figure 2C). A total of 279 genes were classified into subsystem categories using the RAST server and the remaining 393 ORFans were classified manually. The genomic position of the ORFans from complete genomes were extracted and used to generate Supplementary Figure S1. The concatenated protein sequences of the core-genomes of *B. aurantiacum* and *B. linens* complete genomes were used to generate the phylogenetic tree seen in Figure 5. The concatenated sequences of core proteins were aligned using MAFFT (with the flag-auto for the best alignment) (Katoh and Standley, 2013) and the alignment was divided into partitions using the Alignment Manipulation and Summary (AMAS) tool (Borowiec, 2016). Additionally, the best amino-acid substitution model and tree were determined for each partition using IQ-Tree software (Nguyen et al., 2015).

### Insertion Sequence and HGT Identification

ISFinder database was used to identify ISBli1-ISBli5 in the genomes while ISBli6-ISBli35 were identified manually, as follow. We searched for transposase and mobile element annotations with identical lengths, and the corresponding CDS were extracted with 1000 bp upstream and downstream, as previously described (Siguier et al., 2009; Monnet et al., 2010). These sequences were aligned to *Brevibacterium* spp. genomes using BLASTn to confirm the presence of multiple IS copies. When multiple IS copies were identified, inverted repeats were manually annotated. ISs identified manually were submitted to the ISFinder database under the accession name ISBli6 to ISBli35. Four ISs (ISBli18, ISBli20, ISBli22, and ISBli27) were not validated and removed from the dataset. The other IS elements were validated by the ISFinder database. Complete ISs, transposases, and repeat sequences are available in the ISFinder database. To identify putative HGT regions, whole *Brevibacterium* spp. genomes were aligned against the NCBI database using BLASTn. Alignment hits were considered as probable HGT regions when nucleotide sequences larger than 2,000 bp shared more than 90% identity with other bacterial species.

### Prophage Induction Experiments

Prophage induction assays with mitomycin C was performed with six *B. aurantiacum* strains as described elsewhere (Moineau et al., 1994; Capra et al., 2010). *B. aurantiacum* SMQ-1335 and other five *B. aurantiacum* strains (SMQ-1417 – SMQ-1421) were grown in Elliker media with different concentrations of mitomycin C (0.5, 1.0, 2.0, 5.0, and 10 μg/ml) and incubated at 20°C with 200 rpm agitation. The lysogenic strain *Lactobacillus paracasei* A was used as a positive control, while each culture without mitomycin C was used as negative controls.

### Growth Curve With Limited Iron Availability

*Brevibacterium linens* ATCC 19391 and *B. aurantiacum* strains (SMQ-1335, JB5, SMQ-1417 – SMQ-1421) were grown in mineral salts media (MSM) supplemented with trace elements solution (0.53 g/l CaCl_2_, 0.2 g/l FeSO_4_.7H_2_O, 0.01 g/l ZnSO_4_.7H_2_O, 0.01 g/l H_3_BO_3_, 0.01 g/l CoCI_2_.6H_2_O, 0.004 g/l MnSO_4_.5H_2_O, 0.003 g/l Na_2_MoO.2H_2_O, 0.002 g/l NiCl_2_.6H_2_O) (Janssen et al., 1984; Noordman et al., 2006) for 24 h at 30°C, 200 rpm. The cultures were then transferred to fresh MSM in the presence of different concentrations (10, 50, and 100 μM) of the chelating agent ethylenediamine-di-o-hydroxyphenylacetic acid (EDDHA). EDDHA stocks solution (1 mM) was prepared in water (pH 7.0) and filter sterilized. A culture without EDDHA was added as a control to the experiments. Bacterial growth was followed through optical density at 600 nm (OD_600_) for 21 h.

### PCR Testing of BreLI Chromosome Excision

To verify the excision of iRon Uptake/Siderophore Transport Island (RUSTI) (Bonham et al., 2017), we designed an approach targeting the BreLI genomic region based on the strategy described elsewhere (Bonham et al., 2017) (Figure 8). *B. aurantiacum* was cultivated in BHI containing 1% (w/v) agar for 48 h at 30°C and one colony was used for each PCR reaction. PCR was performed using Taq polymerase Mastermix (Feldan) according to the manufacturer’s instructions. All primers used are listed in Table 3. PCR reactions were optimized by adding 10% (v/v) DMSO to the PCR reaction mix. An annealing temperature of 56°C was used to obtain PCR products with a minimum of non-specific bands. Primers 2 and 5 should only form a PCR product if BreLI is excised from the chromosome and is present in a circular form. Sanger sequencing (Plateforme de séquençage et de génotypage des génomes, Quebec, QC, Canada) was performed to confirm BreLI excision.

**Table 3:** List of primers used for the amplification of BreLI.

Primer name	Sequence (5′ → 3′)
1. BreLi1 Fwd	AGTCAGTTGAGATGAGCAGCTG
2. BreLi1 Rev	CTCGATTCTGGTGTTCATGG
3. BreLi2 Fwd	GAACGACCCGATCAACCTGTTC
4. BreLi2 Rev	CTTCTTCAGACTCGGGAATCAG
5. BreLi3 Fwd	CATCGACCGGATGGGAGCTT
6. BreLi3 Rev	ACGTACCTGCAACTTGGAAG

### Production and Activity of Antimicrobial Peptides

*In vitro* production and activity of antimicrobial peptides was tested for actinobacteria and lactic acid bacteria. *B. linens* ATCC 19391, *Corynebacterium glutamicum* ATCC 21086, *B. aurantiacum* SMQ-1417 to SMQ-1421, SMQ-1335, and JB5 were cultivated in Elliker broth at 30°C, 200 rpm. The antimicrobial activity of the filtered supernatants of these strains was tested by spotting 5 μl on a lawn of each of the strains. Additionally, activity was tested against *Micrococcus luteus* HER1157, *C. glutamicum* HER1229, *Arthrobacter arilaitensis* LMA-1184, *Lactococcus lactis* MG1363 and SMQ-1200, *Streptococcus thermophilus* HER1458 and HER1368. The nisin-producing strain *L. lactis* SMQ-1200 was grown in Elliker and incubated overnight at 30°C. This strain was used as a positive control for the experiments against all the bacteria mentioned above.

### Availability of Data and Materials

*Brevibacterium aurantiacum* strains with a SMQ number are available upon request. All IS elements were validated by the ISFinder database under the name ISBli6-ISBli35. Genomes have been deposited in the NCBI genome database (see Table 1 for accession numbers). The complete sequence of pBLA8 was deposited under GenBank accession number CP026735. A list of locus tag identifiers and predicted functions for *B. aurantiacum* ORFans are provided in Supplementary Tables S1A–C. A list of genes in the HGT regions described in this study is provided with their genomic positions and predicted functions in Supplementary Tables S2, S3. All in-house Python and R scripts used in this study are available upon request to the corresponding author.

## Results

### Taxonomic Classification of *B. linens* and *B. aurantiacum*

Our laboratory has access to seven *Brevibacterium* spp. strains, including the dairy strain SMQ-1335 for which its genome was previously sequenced (Melo et al., 2016), five additional dairy strains (SMQ-1417 to SMQ-1421) used in Canada and a strain from the American Type Culture Collection (ATCC 19391) (Table 1). First, we performed their 16S rRNA gene phylogenetic analysis by comparing them with 75 other *Brevibacterium* sequences from GenBank database (NCBI), including type strains for the different species of *Brevibacterium* (Figure 1). With approximately 97% identity, we observed a significant separation between the type strain *B. linens* ATCC 9172 and the six dairy strains from our collection, which were previously believed to belong to the *B. linens* species. Indeed, strain SMQ-1335 as well as the five strains SMQ-1417 to SMQ-1421 grouped with the type strain of *B. aurantiacum* ATCC 9175. Although several other characterized strains (AE038-8, BS258, VKM Ac-2119) have been previously classified as *B. linens*, their 16S rRNA phylogenetic analysis grouped them with the type strain *Brevibacterium epidermidis* (P159), suggesting that these strains should have their taxonomic classification reassessed. Surprisingly, only three strains grouped with *B. linens* type strain ATCC 9172, which are *B. linens* ATCC 19391, VCM10, and Mu101. Only *B. linens* Mu101 and ATCC 9172 were isolated from cheeses. These results suggest that most of the strains used for commercial cheese ripening belong to the *B. aurantiacum* species. For years, *B. linens* has been recognized as a key player on the production of orange-pigmented washed-rind cheeses (Rattray and Fox, 1999; Pham et al., 2017). Whether *B. linens* and *B. aurantiacum* have equal or distinct roles in cheese ripening, remains to be investigated. However, the subdivisions of the *Brevibacterium* species (Gavrish et al., 2004) and new studies involving this group (Cogan et al., 2014; Pham et al., 2017) have highlighted the importance of *B. aurantiacum* in aged cheese production.

### General Genome Features and Plasmid Content

To confirm that our seven *Brevibacterium* strains (SMQ-1335, SMQ-1417 to SMQ-1421, ATCC 19391) were genetically distinct, we first performed PFGE. The seven strains presented a unique PFGE profile using restrictions enzymes AseI and PsiI (data not shown). Then to shed light on their genomic diversity, we sequenced the genomes of six strains (*B. aurantiacum* SMQ-1417 to SMQ-1421, *B. linens* ATCC 19391) using PacBio RSII. Before performing bioinformatic analyses on these six complete genomes, we added to our analyses three other complete genomes (*B. aurantiacum* SMQ-1335 and JB5 as well as *B. epidermidis* BS258), previously sequenced with the same technology (Table 1). The genome length of *B. aurantiacum* strains ranged from 4.035 to 4.424 Mbp with a high G+C content of 62.63 to 62.79% (Table 2). *B. linens* ATCC 19391 and *B. epidermidis* BS258 had smaller genomes (3.862 and 3.807 Mbp) and higher G+C contents (64.16 and 64.79%). The number of predicted coding sequences (CDS) in these two genomes was also lower than in *B. aurantiacum*, correlating with the smaller genome sizes. Four rRNA operons were observed in all *Brevibacterium* strains analyzed. The average number of CDS per *B. aurantiacum* genome was 3,805, of which 79.3% could be assigned a predicted function. We also investigated the presence of CRISPR-Cas system in *B. aurantiacum* or *B. linens* ATCC 19391, but no CRISPR array with the associated Cas genes was predicted in any of the genomes.

The presence of plasmids in *Brevibacterium* is rare with only a few described in the literature (Sandoval et al., 1985; Kato et al., 1989; Ankri et al., 1996; Moore et al., 2003; Nardi et al., 2005; Anast et al., 2019). We searched for the presence of plasmids and only one strain, *B. linens* ATCC 19391, harbored one. The 7,590 bp plasmid named pBLA8 has been described previously and partially sequenced (Leret et al., 1998). We completed pBLA8 sequence (GenBank CP026735) using Illumina MiSeq and identified 16 ORFs. One protein involved in plasmid partitioning and two replication proteins were identified in the theta-replicating plasmid, while the other 13 ORFs have unknown function. We performed a pairwise alignment of pBLA8 with pLIM (7,610 bp), the only other fully sequenced *B. linens* plasmid available (Moore et al., 2003). The alignment revealed that both plasmids are significantly different since the alignment covers only 60% of the longest sequence (88% identity). Interestingly, different regions from pBLA8 were found in five *B. aurantiacum* genomes (≥99% identity), suggesting genetic exchange between these two closely related species (Supplementary Figure S2). These genomic regions could also correspond to an ancestral version of pBLA8 present in a common *Brevibacterium* ancestor that became unable to replicate autonomously. Up to 80% of the plasmid was found into different regions of the genomes of *B. aurantiacum* SMQ-1417 and SMQ-1335, including regions coding for RepA and RepB. The only plasmid regions absent in these two bacterial genomes were the origin of replication *(ori)* and genes encoding an additional non-essential replication protein and a hypothetical protein (ORF16). Of note, we found orthologs of JetA, JetB, JetC, and JetD of the recently described anti-plasmid system Wadjet (Doron et al., 2018) in the genome of *B. linens* ATCC 19391 and *B. epidermidis* BS258 as well as in all seven *B. aurantiacum* strains studied here. Although other unknown systems could be involved, this anti-plasmid system could be partly responsible for the absence of plasmids in *B. aurantiacum* strains analyzed here.

### Core-Genome and Pan-Genome

To determine the genetic diversity of *B. aurantiacum*, we performed a pan-genome analysis. In addition to the seven *B. aurantiacum* complete genomes now available, we added four *B. aurantiacum* draft genomes recently sequenced with a different technology (Pham et al., 2017). Our analysis revealed that the open pan-genome for 11 *B. aurantiacum* strains reaches 6,259 genes (Figure 2A). We also analyzed the number of genes present in different number of *k* genomes. The two major groups composed the core genome (*k* = 11 genomes) and the orphan genes (*k* = 1 genome), with 2,612, and 1,790 genes, respectively (Figure 2B). Hence, most of *B. aurantiacum* genes are either conserved in all genomes or specific to one strain (Figure 2B). The orphan genes/proteins, also called ORFans, increase the size of *B. aurantiacum* pan-genome. To explore the functions of these orphan genes, we annotated and classified them into categories using RAST. Manual curation was also performed for proteins that were not classified by RAST (Supplementary Tables S1A–C). Of 1,790 ORFans, 886 (49.5%) were hypothetical proteins. From the remaining, 125 (7.0%) had putative functions, 100 (5.6%) were considered miscellaneous, and 679 proteins (37.9%) were classified into RAST subsystem categories (Figure 2C). In RAST, phage and mobile element proteins had 112 representatives, which represented the main functional categories of ORFans, suggesting that *B. aurantiacum* contains a diverse array of mobile genetic elements. Proteins involved in membrane transport (104 genes), DNA and RNA metabolism (99 genes), and transcriptional regulators (83 genes) also accounted for several ORFans. A total of 20 Type I restriction-modification (R-M) systems and 6 Type III R-M methylation subunits were identified in the DNA and RNA metabolism category, perhaps offering broad protection against phage infection. Another intriguing category among *B. aurantiacum* ORFans was ATP-Binding Cassette (ABC) transporter proteins, such as components of iron siderophore transport system. In the cheese environment where iron is poorly available, these genes could improve growth on cheese rinds. Furthermore, we analyzed the genomic context of the ORFans and found that these genes were dispersed into *B. aurantiacum* genomes (Supplementary Figure S1). A slightly higher concentration of ORFans was observed 2.5 Mbp downstream of the *ori* region (upstream *dnaA*) in all eleven *B. aurantiacum* genomes suggesting that this chromosomal region is less conserved in this species.

### Transposable Elements

After observing parts of pBLA8 and several mobile elements in the *B. aurantiacum* pan-genome, we analyzed the *B. aurantiacum* mobilome. In the largest *B. aurantiacum* genome (strain SMQ-1417), we identified 116 transposases and 68 integrases indicating that this species contains several mobile genetic elements, such as prophages and insertion sequences (IS). A putative prophage was previously identified in the genome of SMQ-1335 (Melo et al., 2016). Although the presence of several transposases in this putative prophage suggested that it was not functional, we tested SMQ-1335 and the other *B. aurantiacum* strains from our collection for the presence of inducible prophages. Even though the cultures with mitomycin C grew slower than the control without the chemical, no significant drop on bacterial optical density was observed for *B. aurantiacum* cultures. These results suggested that these strains did not carry functional prophages in their genome or that they could not be induced in the conditions tested.

Once the genome was annotated, we observed genes encoding putative phage proteins (e.g., holin, portal, major capsid protein, tail tape measure protein) spread in the genomes of all six *Brevibacterium* strains recently sequenced, suggesting the presence of prophage remnants. We then used *in silico* analyses to detect putative prophages throughout the genomes of *B. linens* ATCC 19391 and *B. aurantiacum* SMQ-1417 – SMQ-1421. Similar to SMQ-1335, several integrases, transposases and other mobile elements were inserted within prophage-like regions, suggesting that they were likely inactivated overtime. Of note, a seemingly incomplete prophage (27.3 kbp) was detected in the genome of *B. aurantiacum* SMQ-1419. Although no structural gene was predicted, we performed a manual inspection of the regions flanking the putative prophage and found a gene encoding a putative tail tape measure protein (TMP) located a few ORFs upstream of PHASTER predicted attachment site *attL*. The BLASTp analysis of other ORFs surrounding the TMP also showed the presence of other genes involved in phage morphogenesis, such as putative genes coding for minor tail proteins, tail assembly chaperone and major capsid protein. We also identified genes involved in phage DNA packaging (e.g., portal and terminase). This extended 45-kbp putative prophage in SMQ-1419 was not found in other *B. aurantiacum* strains.

**FIGURE 3:**
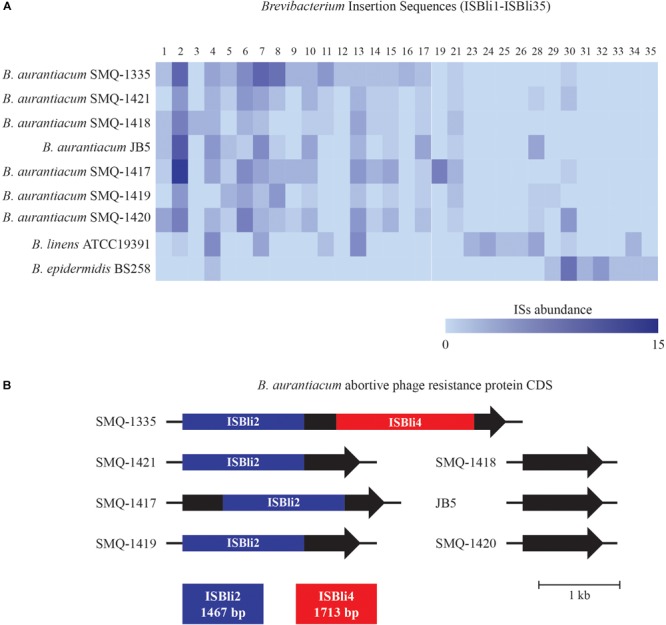
*Brevibacterium* Insertion Sequences (ISBli1-ISBli35). **(A)** Abundance of ISs in *B. aurantiacum* and *B. linens* genomes. ISBli1-ISBli5 were identified with the ISFinder database and ISBli6-ISBli35 were identified manually. **(B)** Schematic representation of the integration of ISBli2 and ISBli4 in the coding region of an abortive phage resistance protein CDS in *B. aurantiacum* genomes. Coding sequences correspond to locus tags BLSMQ_RS02885 – BLSMQ_RS02895 of *B. aurantiacum* SMQ-1335 (GenBank: NZ_CP017150.1).

As part of *B. aurantiacum* mobilome, IS also play an important role in bacterial evolution by spreading into a genome and creating genetic variations (Wright et al., 2016). They can inactivate genes when integrated into a coding region or a promoter region as well as activate gene expression by providing an alternative promoter (Vandecraen et al., 2017). Moreover, neighboring genes can be translocated by two flanking IS in a so-called composite transposon (Varani et al., 2015; Vandecraen et al., 2017). Using the ISFinder database, we detected five ISs (ISBli1-ISBli5) in *B. aurantiacum* genomes, often in several copies. Manually curating the annotated genomes led to the identification of 14 additional types of ISs in *B. aurantiacum* and 12 distinct in *B. linens* (ISBli6-ISBli35). A total of 26 ISs were validated by the ISFinder database, whereas four (ISBli18, ISBli20, ISBli22, and ISBli27) were not validated for not being discriminating enough to the others and were then removed from our analysis. We observed a dichotomy in the abundance and the type of ISs detected in *B. aurantiacum* and *B. linens* genomes (Figure 3A), suggesting a role in the diversification of these two closely related species. While a total of 56 copies of ISBli2 were identified in all seven *B. aurantiacum* genomes (5 to 13 copies/genome), only one copy was identified in *B. linens* ATCC 19391.

Interestingly, we found ISBli2 integrated into a gene coding for a putative abortive phage resistance protein in the genomes of *B. aurantiacum* SMQ-1417, SMQ-1419, SMQ-1421, and SMQ-1335 (Figure 3B). In SMQ-1335, the same gene was also disrupted by ISBli4. This putative anti-phage gene was identified in all *B. aurantiacum* genomes but was intact in *B. aurantiacum* SMQ-1418, SMQ-1420, and JB5. Therefore, we hypothesize that ISBli2 and ISBli4 may have inactivated this phage resistance protein in four out of seven *B. aurantiacum* genomes. Bacteriophages (or simply phages) infecting dairy cultures are well documented (Marcó et al., 2012; de Melo et al., 2018) and the presence of an intact abortive phage infection protein could provide protection against *Brevibacterium* phages during cheese ripening.

**FIGURE 4:**
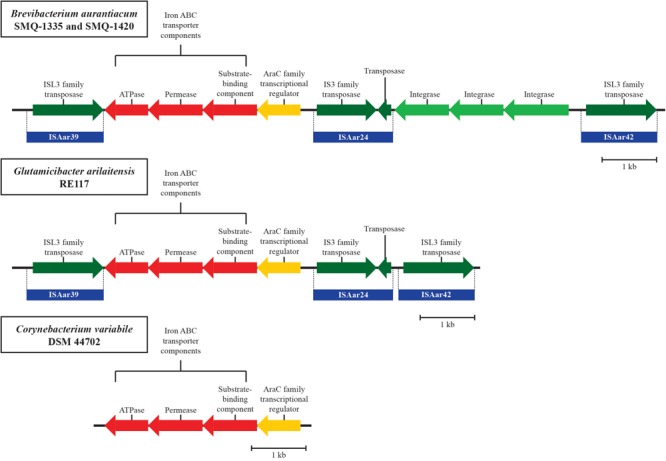
Schematic representation of the iron uptake composite transposon identified in *B. aurantiacum* SMQ-1335 and SMQ-1420. The homologous iron uptake gene clusters found in *G. arilaitensis* RE117 and *Corynebacterium variabile* DSM 44702 are also illustrated. Genes involved in iron transport are shown in red and the AraC family transcriptional regulator is shown in yellow. Mobile element proteins are shown in green and the ISs from *G. arilaitensis* are shown in blue. CDS correspond to locus tags BLSMQ_RS13730 - BLSMQ_RS13785 of *B. aurantiacum* SMQ-1335 (GenBank: NZ_CP017150.1).

We found ISs from two other actinobacteria species used for cheese production in *B. aurantiacum* genomes. We identified ISPfr2, from *Propionibacterium freudenreichii*, in all *B. aurantiacum* strains. ISAar24, ISAar39, and ISAar42 from *G. arilaitensis* were also identified, but only in *B. aurantiacum* SMQ-1335 and SMQ-1420. The presence of these IS elements strongly suggests genetic exchange between cheese rind actinobacteria (Bonham et al., 2017). In-depth analysis of the 11,797 bp region containing homologs of the *G. arilaitensis* ISs also revealed an iron uptake gene cluster between ISAar24 and ISAar39 (Figure 4) in both *B. aurantiacum* strains. *B. aurantiacum* iron uptake gene cluster shares 99% nucleotide identity with the one found in the strain *G. arilaitensis* RE117. ISAar39 and ISAar42 flanks the 11,797 bp genomic region and contain ISL3 family transposases sharing 65% amino acid sequence identity. The right inverted repeats of these two IS share 73.2% nucleotide sequence identity. Therefore, this region could be a composite transposon. Of note, the three integrase genes between ISAar24 and ISAar42 in *B. aurantiacum* SMQ-1335 and SMQ-1420 are absent in *G. arilaitensis* RE117, suggesting that their presence in *B. aurantiacum* occurred after the integration of the composite transposon (Figure 4). Interestingly, the iron uptake/siderophore operon was also identified in *Corynebacterium variabile* DSM 44702 (99% identity), but without the IS (Figure 4). We hypothesize that this operon originated from this species before being mobilized between other cheese rind actinobacteria.

### Horizontal Gene Transfer Regions

**FIGURE 5:**
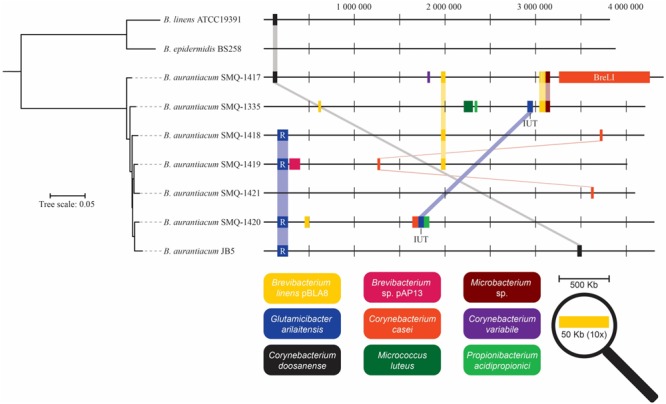
Schematic representation of HGT regions identified in *Brevibacterium* genomes. Homologous DNA sequences present in different species were identified with BLAST alignments. Identical HGT regions present in different genomes are linked together. HGT region sizes are increased 10× for presentation purposes and only regions with >90% identity are shown. The phylogenetic tree was generated with the concatenated protein sequences of the core-genomes of *B. aurantiacum, B. linens*, and *B. epidermidis*. R, iRon Uptake Siderophore/Transport Island (RUSTI); IUT, Iron Uptake Transposon; BreLI, *Brevibacterium* Lanthipeptide Island.

While performing BLAST analysis, we noticed that all *B. aurantiacum* genomes contained homologous nucleotide sequences (92–99% identity) with other species of cheese rind actinobacteria (Figure 5). Genomic positions, description, and gene annotations of these putative HGT regions are provided in Supplementary Tables S2, S3. An HGT region (12,314 bp) from the previously described RUSTI (Bonham et al., 2017) was identified in four *B. aurantiacum* genomes (Figure 5). The largest HGT region (99,628 bp) was observed in SMQ-1417 and shared 99% identity over 96% of its length with *C. casei* LMG S-19264. This region has been previously described as the *Brevibacterium* Lanthipeptide Island (BreLI) (Walter et al., 2014; Pham et al., 2017). It is noteworthy that only two genes related to lanthipeptides synthesis has been assigned a function in the HGT region found in the strains from our collection.

**FIGURE 6:**
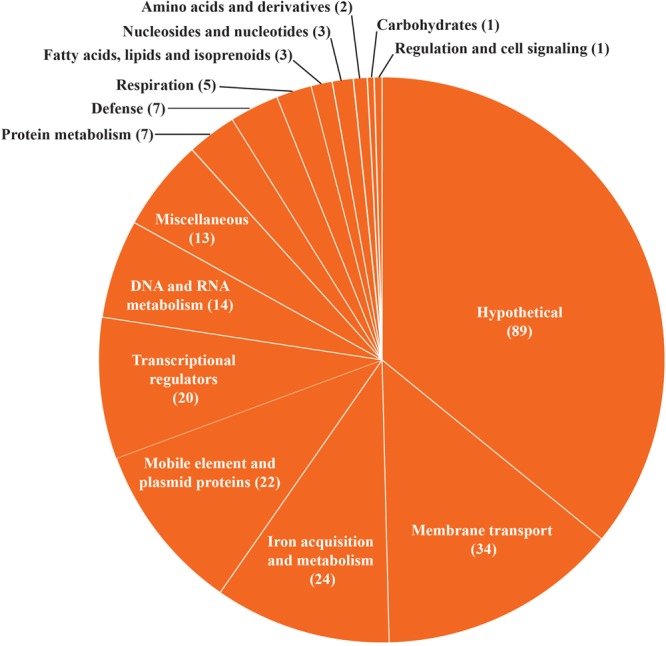
Functional classification of *B. aurantiacum* horizontally transferred genes. Similar metabolic subsystem categories are fused together and the percentage of HGT genes in each RAST subsystem category is shown.

We extracted annotations from all HGT regions identified in *B. aurantiacum* genomes and clustered them into functional groups based on the RAST subsystem categories (Figure 6). For representation purposes, we combined similar metabolic subsystem categories. The hypothetical proteins category contains the highest number of genes (89). The mobile elements (transposases, integrases, etc.) and plasmid protein category contains 22 genes. We also identified a total of 34 genes involved in membrane transport and 24 genes involved in iron acquisition and metabolism. Moreover, 7 of the 20 transcriptional regulators observed in the HGT regions correspond to the AraC family. This family of transcriptional regulators is often found in the iron uptake gene clusters (Bonham et al., 2017). This observation suggests that efficient transport systems could confer a selective growth advantage to *B. aurantiacum* strains by helping them to efficiently acquire nutrients and minerals from the cheese environment. Of note, genes involved in DNA mobilization, membrane transport and iron acquisition were also the most prevalent horizontally transferred genes previously observed in 165 cheese rind bacteria (Bonham et al., 2017). Taken altogether, these observations suggest high rates of HGT between cheese rind actinobacteria and cooperative adaptation to the cheese surface.

### Iron Uptake and Siderophore Synthesis

Efficient iron acquisition systems are a driving force in adaptation to the iron-depleted cheese surface (Monnet et al., 2012; Bonham et al., 2017). Iron acquisition is mediated by siderophores, which are secreted by bacteria in response to iron depletion and act as chelating agents to scavenge iron (Andrews et al., 2003). In Gram-positive bacteria, iron uptake is mediated by three components: a membrane-anchored binding protein, permease, and ATP-binding protein cassette (Andrews et al., 2003). Different patterns of siderophore production and utilization in *Brevibacterium* spp. were previously described and some strains were shown to be auxotrophic for iron siderophores (Noordman et al., 2006). Hence, the addition of siderophores or co-cultivation of siderophore-producing strains with auxotrophic strains can stimulate the growth of the latter (Noordman et al., 2006). To study the influence of the iron metabolism on *B. aurantiacum*, we analyzed the predicted genes involved in iron siderophores synthesis and acquisition. Iron ABC transporter components were more abundant in *B. aurantiacum* SMQ-1418, SMQ-1419, SMQ-1420, and JB5 than in the other strains (Supplementary Table S4). The genome of *B. aurantiacum* JB5 harbors 26 genes linked to iron uptake, the highest number in the set of genomes analyzed in this study.

Three iron ABC transporter components are clustered together in *B. aurantiacum, B. linens*, and *B. epidermidis* genomes. In addition to the two clusters originating from the RUSTI region and the iron uptake transposon, three different iron uptake gene clusters were identified in the genome of all seven *B. aurantiacum* strains from our collection. A putative hydroxamate-type siderophore biosynthesis cluster encoding a siderophore synthetase, a lysine N6-hydroxylase, and a L-2,4-diaminobutyrate decarboxylase was recently described (Pham et al., 2017). Excluding *B. linens* ATCC 19391, this siderophore gene cluster was identified in *B. epidermidis* BS258 and all seven *B. aurantiacum* genomes. The additional putative catecholate siderophore gene cluster (Pham et al., 2017) was also observed in *B. aurantiacum* SMQ-1420 and JB5. Thus, all dairy strains from our dataset seem to be able to produce iron siderophore.

**FIGURE 7:**
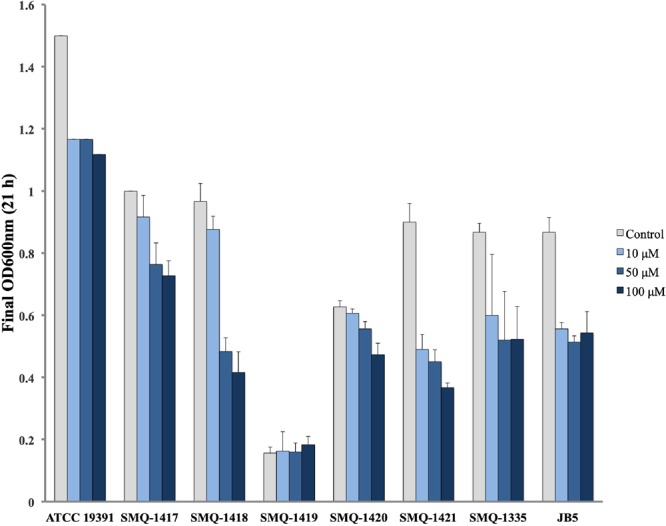
Growth of *Brevibacterium* spp. in iron-limited conditions. The final optical density at 600 nm after 21 h growing in the presence or absence of EDDHA (0, 10, 50, or 100 μM) is shown in the graph for *B. linens* ATCC 19391 and *B. aurantiacum* strains.

Considering the diverse amount of iron uptake gene clusters observed in *B. linens* ATCC 19391 and *B. aurantiacum* strains (SMQ-1335, JB5, SMQ-1417 to SMQ-1421), we performed growth curves in a MSM with limited iron availability and the presence of increasing concentrations of EDDHA as a chelating agent (Figure 7). We hypothesized that the acquisition of horizontally transferred iron uptake genes would confer growth fitness in an iron-limited media. SMQ-1419 did not grow well in MSM under the conditions tested, which can be either related to iron availability or another growth requirement missing in the media for this strain. In general, bacterial growth was inversely proportional to the concentration of EDDHA in the media, but the strains grew in presence or absence of chelator. In comparison with the *B. aurantiacum* strains, our experiments showed that *B. linens* ATCC 19391 is better adapted to iron-limited conditions, growing well in all concentrations of chelating agent tested. It has been previously shown (Noordman et al., 2006) that 100 μM EDDHA is a stringent condition for the growth of siderophore-auxotrophic strains, however, it is likely less strict than in the cheese habitat. In stringent condition, SMQ-1417 grew better among *B. aurantiacum* strains, reaching approximately an OD_600_ of 0.7 after after 21 h in the conditions tested. On the other hand, SMQ-1421 grew the least and also presented a larger growth difference between the control (without EDDHA) and cultures with the addition of chelating agent. Since SMQ-1421 is the strain with the lower number of iron-uptake components, this result supports the premise that the presence of genes/proteins cluster involved in iron acquisition is likely to confer adaptive growth on the cheese surface. However, they are not directly proportional as can be seen with JB5, which is the strain with more iron acquisition components. JB5 presented a similar growth in all three EDDHA concentrations, but not as much as the control culture that grew faster and reached a higher optical density. These results suggest that most of *Brevibacterium* strains are adapted to the growth in iron-limited medium.

### *Brevibacterium* Lanthipeptide Island (BreLI)

Another factor that can influence the microbial community on cheese surface is the production of antimicrobials, such as bacteriocins. For example, lanthionine-containing peptides (lanthipeptides) can inhibit the growth of undesirable microorganisms (Gomes et al., 2017) and confer a selective advantage to the producing cultures. These antimicrobials are usually synthesized as inactive precursor peptides that undergo post-translational modifications to generate active molecules (Gomes et al., 2017). A probable Integrative Conjugative Element (ICE) coding for a lanthipeptide synthesis gene cluster has been previously identified in six cheese-associated *Brevibacterium* strains (*B. antiquum* CNRZ 918 and P10, *B.* aurantiacum ATCC 9174 and CNRZ 920, *B. linens* ATCC 9172 and Mu101) (Pham et al., 2017). This 96–100 kbp genomic island, called *Brevibacterium* Lanthipeptide Island (BreLI), was also observed in *C. casei* LMG S-19264 (Walter et al., 2014). This ICE integrated at the 3′ end of a gene encoding a class Ib ribonucleotide reductase beta subunit, which resulted in 12 bp repeat sequence (5′-AGAAGTCCCAGT-3′) flanking each side of BreLI (Pham et al., 2017). We identified this genomic island in the genome of *B. aurantiacum* SMQ-1417. ICE elements can be identified by the presence of signature genes/proteins associated with the core conjugation functions of (i) integration and excision from the host genome, (ii) replication as an extrachromosomal element, and (iii) conjugation between host and recipient cells (Ghinet et al., 2011). Genes involved in all three core functions were identified in *B. aurantiacum* SMQ-1417 BreLI region. An integrase (CXR23_14705) and a site-specific integrase (CXR23_15110) were observed at the two borders of BreLI, directly beside the repeats. Two other integrases (CXR23_14820, CXR23_14825), and one excisionase (CXR23_14930) were also identified and these five genes are likely involved in the integration and excision of BreLI.

**FIGURE 8:**
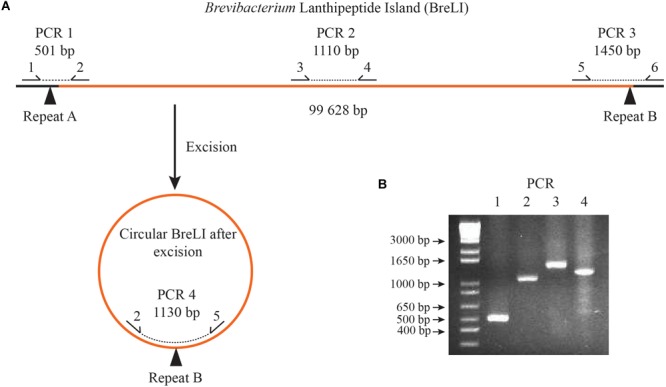
PCR amplification of *B. aurantiacum* SMQ-1417 BreLI. **(A)** Schematic for PCR primer design. **(B)** PCR testing for the presence of BreLI and for the excision of the ICE from the chromosome of *B. aurantiacum* SMQ-1417. PCR products were migrated on a 2% agarose gel for 35 min at 110 volts and the gel was stained with ethidium bromide before UV observation.

To our knowledge, no experiment had been previously performed to determine whether this ICE element is active. To confirm excision from the genome, we designed PCR primers to amplify different regions of the *B. aurantiacum* SMQ-1417 ICE element (Figure 8). When combined, primers 2 and 5 were expected to generate a PCR product only if BreLI was excised from the chromosome. The detection and sequence of the PCR products confirmed the excision of BreLI and its presence in a circular form (Figure 8). Thus, this ICE element could still excise from the *B. aurantiacum* SMQ-1417 chromosome and potentially perform conjugation.

In terms of DNA replication, *B. aurantiacum* SMQ-1417 BreLI contains two helicases (CXR23_14715, CXR23_15070), a DNA polymerase III subunit alpha (CXR23_14790) and a DNA primase (CXR23_14925). The majority of actinobacterial ICEs utilizes a FtsK homolog-based conjugative DNA-translocation system to exchange double-stranded DNA (Bordeleau et al., 2012). A conjugal transfer protein (CXR23_14800), a chromosome partitioning protein ParB (CXR23_14945), a conjugal transfer protein TrbL (CXR23_14995), a type VI secretion protein (CXR23_15060), and a single-stranded DNA-binding protein (CXR23_15075) are also present in BreLI. These results suggest that BreLI uses a single-stranded DNA (ssDNA) transfer system. Interestingly, the *G. arilaitensis* JB182 RUSTI, observed in four *B. aurantiacum*, is also believed to use a ssDNA transfer mechanism (Bonham et al., 2017).

We used Bagel4 to find genes involved in the biosynthesis of bacteriocins or bactericidal post-translational modified peptides in the genome of *B. aurantiacum* strains and *B. linens* ATCC 19391. *B. linens* ATCC 19391 and all five *B. aurantiacum* strains sequenced here as well as SMQ-1335 contain a gene encoding a homolog of linocin. SMQ-1417 and ATCC 19391 possess a gene cluster involved in the synthesis of the lanthipeptide NAI-112, including a homolog for the core peptide. SMQ-1417, SMQ-1418, and SMQ-1419 have also genes involved in corynazolicin synthesis (e.g., core peptide) and linaridin modification and transport, whereas SMQ-1420 and SMQ-1421 only have the genes involved in biosynthesis. SMQ-1335, SMQ-1418, and SMQ-1419 contain a putative gene cluster involved in sactipeptide production, although they do not encode putative antimicrobial peptide precursors.

We tested the *in vitro* antimicrobial activity of *Brevibacterium* spp. filtered supernatant against different bacterial species. We used the class I lantibiotic nisin-producing strain *L. lactis* SMQ-1220 as a positive control in our experiments. Although nisin-containing supernatants of SMQ-1220 were active against the bacterial strains tested (see section “Materials and Methods,” for the list), we did not observe antimicrobial activity for the filtered supernatants of *Brevibacterium* strains in the conditions tested. Perhaps, the lack of activity is related to experimental parameters, but it can also be due to genetic features of the host cells that prevent the expression of the laterally shared genes, such as promoter incompatibilities (Ochman et al., 2000) or absence of some components that act in the post-translational modification of the peptide (Gomes et al., 2017).

## Discussion and Conclusion

Comparative genomic studies have led to a better understanding of the genetic adaptation of dairy bacteria, such as *S. thermophilus* (Goh et al., 2011), *L. lactis* (Kelleher et al., 2017), *C. variabile* (Schröder et al., 2011), *G. arilaitensis* (Monnet et al., 2010), and *P. freudenreichii* (Falentin et al., 2010) to cheese. A recent comparative analysis of mostly draft genomes of 23 *Brevibacterium* strains also provided insights into their adaptation to the cheese habitat (Pham et al., 2017). Overall, these studies shed light on the evolution and role of each bacterium in the development of cheese aromas and flavors.

Given the prevalence of mobile genetic elements in *Brevibacterium* spp. and other cheese actinobacteria (Bonham et al., 2017; Pham et al., 2017), we used PacBio long read sequencing technology to obtain six new complete *Brevibacterium* genome sequences and added information on the mobilome of these bacteria. Our phylogenetic analysis support previous findings (Cogan et al., 2014; Pham et al., 2017) and shows that most *Brevibacterium* strains used for cheese production belong to the *B. aurantiacum* species, making this species a key player in the dairy industry. Therefore, the taxonomic position of commercial *Brevibacterium* strains should be revisited.

We used environmental strains of *B. linens* and *B. epidermidis* to compare with *B. aurantiacum* dairy strains and understand the cheese domestication of *B. aurantiacum*. Despite being the most prevalent (Cogan et al., 2014; Dugat-Bony et al., 2016), *B. aurantiacum* is not the only *Brevibacterium* species able to grow on cheese. For example, *B. antiquum, B. casei*, and *B. linens* have been isolated from cheeses (Monnet et al., 2015; Pham et al., 2017). However, certain genetic traits may explain the prevalence of *B. aurantiacum* in the dairy ecosystem. Multiple HGTs have been documented between cheese rind bacteria (Bonham et al., 2017) and between cheesemaking fungi (Cheeseman et al., 2014), suggesting a complex network of gene exchanges that are shaping the evolution and adaptation of cheese-associated microorganisms. Our comparative analyses found significant differences between the mobilome of *B. aurantiacum* dairy strains and environmental strains of *B. linens* and *B. epidermidis*. *B. aurantiacum* strains seem to have acquired heterologous genes from other dairy actinobacteria, such as *G. arilaitensi*s, *C. variabile*, and *C. casei*. The high percentage of identity between these DNA segments suggests that these genes were recently transferred. Moreover, mobile genetic elements are expanding *B. aurantiacum* pan-genome and they contribute to its genetic diversity. It is noteworthy that *B. aurantiacum* pan-genome is still open and more genomes should be analyzed to appreciate the diversity of its gene repertoire.

The use of commercial ripening cultures with active mobile genetic elements could explain the prevalence of nearly identical DNA sequences between cheese rind actinobacteria (Bonham et al., 2017), such as the lanthipeptide genomic island present in *Brevibacterium* spp. genomes. PCR amplifications of BreLI confirmed that this ICE was still active in *B. aurantiacum* SMQ-1417. Although *in vitro* tests did not confirm antimicrobial activity, it is still likely that an efficient production of active lanthipeptides could confer a selective advantage for the producing strain. Also observed in *C. casei* LMG S-19264, BreLI is likely to have been mobilized from a *Brevibacterium* spp., perhaps during cheese ripening (Walter et al., 2014). With these observations, one can speculate that as a species commonly used in commercial ripening cultures, *B. aurantiacum* is likely a player in the widespread distribution of mobile genetic elements.

Here, we found horizontally transferred regions involved in iron uptake in five out of seven *B. aurantiacum* genomes. As previously showed (Bonham et al., 2017), genes associated with iron uptake were the most common genes exchanged between cheese actinobacteria. To assess if strains with efficient iron uptake systems are likely more adapted to the iron-depleted cheese surface, we performed growth curves of *Brevibacterium* strains in media with limited iron availability. Except with SMQ-1419, that was not able to grow in the media, all the other strains grew in increasing amount of iron chelating agent, with variability in the growth pattern. The strain SMQ-1421 was the less fit in iron-limited media, which corroborates with the lower abundance of iron acquisition and siderophore biosynthesis genes. On the other hand, the environmental strain *B. linens* ATCC 19391 was the better adapted when growing in the presence or absence of chelating agent.

Growth on the surface of smear-ripened cheeses is dependent of the capability to use substrates available in the cheese habitat. Additionally, it depends on its interactions with members of the microbial community present during ripening. Further studies could be done to explore the capacity of different *Brevibacterium* strains to grow, alone or in combination, in iron-depleted environments. Some *B. aurantiacum* strains with high aromatic potential could be auxotrophic for iron siderophores and dependent on other strains or species to grow well on cheese. Cheesemakers are sometimes faced with unstable ripening activity or the growth of undesirable microorganisms (Monnet et al., 2016). The optimization of ripening cultures while addressing these issues may improve the production of high-quality smear-ripened cheeses.

In conclusion, the addition of six complete genomic sequences of *Brevibacterium* spp. allowed an in-depth analysis of the mobilome of commercial *B. aurantiacum* strains and complement previous comparative analysis targeting cheese-related actinobacteria. Our phylogenetic analysis demonstrated that the industrial strains used for cheese production belong to the *B. aurantiacum* species. Our study also revealed that mobile genetic elements are widespread among the strains analyzed here and contribute to *B. aurantiacum* genetic diversity. Moreover, iron uptake HGT demonstrate the cooperative evolution of cheese actinobacteria, allowing their niche adaptation to the iron-depleted cheese surface. Regardless of the strong selective pressure exerted on the surface of smear-ripened cheeses, *B. aurantiacum* strains seem to be well adapted to thrive in this ecological niche. This comprehensive genomic information will serve as a tool to continue improving our understanding of the complex interactions taking place in smear-ripened cheese microbial communities.

## Contribution to the Field

Only two complete *B. aurantiacum* genomes were available in public databases prior to this study. *B. aurantiacum* genomes contain a high number of transposable elements and their genome sequences remain at the draft level when sequenced with short-read sequencing technologies, which precludes in-depth analysis of mobile genetic elements and genome plasticity. Here, we used long-read sequencing technology to provide six new complete *Brevibacterium* genomes. We identified a high number of mobile genetic elements that could be involved in the growth fitness of *B. aurantiacum* on the surface of smear-ripened cheeses. These genetic traits are shared between cheese rind actinobacteria and they seem involved in iron acquisition. Our study shows that mobile genetic elements are widespread in *B. aurantiacum* and contribute to its genomic diversity.

## Data Availability

The datasets generated for this study can be found in GenBank, CP025330, CP025331, CP025332, CP025333, CP025334, and CP026734.

## Author Contributions

SL and SM designed the research protocol. SL, AM, and SJL performed the research. SJL wrote the in-house python and R scripts and performed the pan/core-genome analysis. SL and AM analyzed the data. SL, AM, and SM wrote the manuscript.

## Conflict of Interest Statement

The authors declare that the research was conducted in the absence of any commercial or financial relationships that could be construed as a potential conflict of interest.
