# Supplementary material for: Mobilome of Brevibacterium aurantiacum Sheds Light on Its Genetic Diversity and Its Adaptation to Smear-Ripened Cheeses
Source: Front Microbiol. 2019 Jun 10;10:1270. doi: 10.3389/fmicb.2019.01270 (PMC6579920; doi:10.3389/fmicb.2019.01270)
Supplement: Supplementary file 2 [file Table_2.DOCX]

**Supplementary Table S2 Horizontal gene transfer (HGT) regions identified in *B. aurantiacum* and *B. linens* genomes.** HGT regions were identified with NCBI BLAST alignment and only region with >90% identity are shown. Every gene annotations and original genomic positions are presented in additional file 3. Gene annotations and genomic positions are presented in Supplementary Table S3.

| **BLAST Hit** | **HGT region length (bp)** | **Identity**  **(%)** | **Strains** | **Genomic positions** | **HGT region description and features** |
| --- | --- | --- | --- | --- | --- |
| ***Glutamicibacter arilaitensis* RE117** | 6875 | 99 | SMQ-1335  SMQ-1420 | 2990313 – 2997187  1756202 – 1763075 | Iron uptake composite transposon |
|  | 12 314 | 94 | SMQ-1420  SMQ-1419  JB5  SMQ-1418 | 162898 – 175210  162006 – 174318  183569 – 195883  170459 – 182771 | Iron Uptake/Siderophore Transport Island (RUSTI) |
| ***Corynebacterium casei***  **LMG S-19265** | 5671 | 97 | SMQ-1420 | 1745627 – 1751286 | - Directly upstream of the Iron uptake composite transposon  - Code for a DNA-methyltransferase, a D-lactate dehydrogenase and a ISL3 family transposase |
|  | 2401 | 97 | SMQ-1419  SMQ-1418  SMQ-1421 | 1164816 – 1167215  3756874 – 3759273  3651273 – 3653672 | Code for an hypothetical protein and a ltrA CDS |
|  | 100 186 | 99 (96% coverage) | SMQ-1417 | 3165461 – 3265646 | - BreLi island  - Truncated by mobile genetic elements  - Code for 83 genes, including ABC transporters, bacteriocin (lantipeptide), transposases, integrases, conjugal transfer proteins and other mobile element proteins (See additional file 3 for more information) |
| ***Acidopropionibacterium acidipropionici* ATCC 4875** | 5852 | 92 | SMQ-1420 | 1764236 - 1770082 | - Directly downstream of the Iron uptake composite transposon  - Code for a recombinase, an antirestriction protein ArdA and 7 hypothetical proteins |
|  | 2965 | 99 | SMQ-1335 | 2227500 – 2230464 | Code for a recombinase, a transposase and an ATP-binding protein |
| ***Corynebacterium variabile* DSM44702** | 2862 | 95 | SMQ-1417 | 1821567 – 1824418 | Code for an oleate hydratase, a recombinase and a TetR family transcriptional regulator |
| ***Brevibacterium linens***  **ATCC 19391 plasmid pBLA8** | 2688 | 99 | SMQ-1335 | 586281 – 588969 | Code for hypothetical plasmid proteins |
|  | 5767 | 99 | SMQ-1335 | 3076421 – 3082188 | Code for plasmid replication proteins (RepA, RepB) and hypothetical proteins |
|  | 3 832 | 99 | SMQ-1417  SMQ-1418  SMQ-1419 | 2055761 – 2059591  1931719 – 1927889  1899939 – 1896109 | Code for a plasmid replication protein (RepB) and hypothetical proteins |
|  | 5 771 | 99 | SMQ-1417 | 3108612 – 3111809  3118264 – 3118522  3120079 – 3120874  3122305 – 3123805 | - Code for plasmid replication proteins (RepA and RepB) and hypothetical proteins  - Truncated by mobile elements |
|  | 4 926 | 99 | SMQ-1420 | 346511 – 351436 | Code for plasmid replication proteins (RepA and RepB) and hypothetical proteins |
| ***Brevibacterium* sp. plasmid pAP13** | 11 856 | 96 | SMQ-1419 | 228446 – 240302 | Code for a sodium-independent anion transporter, a MFS transporter, a ribonuclease, a MerR family transcriptional regulator and hypothetical proteins |
| ***Microbacterium* sp. No. 7** | 5 284 | 96 | SMQ-1417  SMQ-1335 | 3123844 – 3129112  3082210 – 3087479 | Code for a chromate transporter,  an heavy metal translocating P-type ATPase and a vacuolar iron-transporter (VIT) family protein |
| ***Micrococcus luteus* NCTC 2665** | 8 337 | 96 | SMQ-1335 | 2204340 – 2212655 | Code for a nucleotide-binding protein, a thioredoxin, an heat shock protein transcriptional repressor HspR, a nucleotide exchange factor GrpE and molecular chaperones ClpB, DnaJ and DnaK |
| ***Corynebacterium doosanense* CAU212** | 6704 | 99 | SMQ-1417  JB5  ATCC 19391 | 176411 – 183114  3495872 – 3502576  142288 – 148990 | Code for a MFS transporter, a 3-oxoacyl-ACP reductase, two NUDIX domain-containing proteins, a DNA-methyltransferase, a thymidylate synthase and a nucleoside 2-deoxyribosyltransferase |
