# Supplementary material for: Mobilome of Brevibacterium aurantiacum Sheds Light on Its Genetic Diversity and Its Adaptation to Smear-Ripened Cheeses
Source: Front Microbiol. 2019 Jun 10;10:1270. doi: 10.3389/fmicb.2019.01270 (PMC6579920; doi:10.3389/fmicb.2019.01270)
Supplement: Supplementary file 5 [file Data_Sheet_1.docx]

**
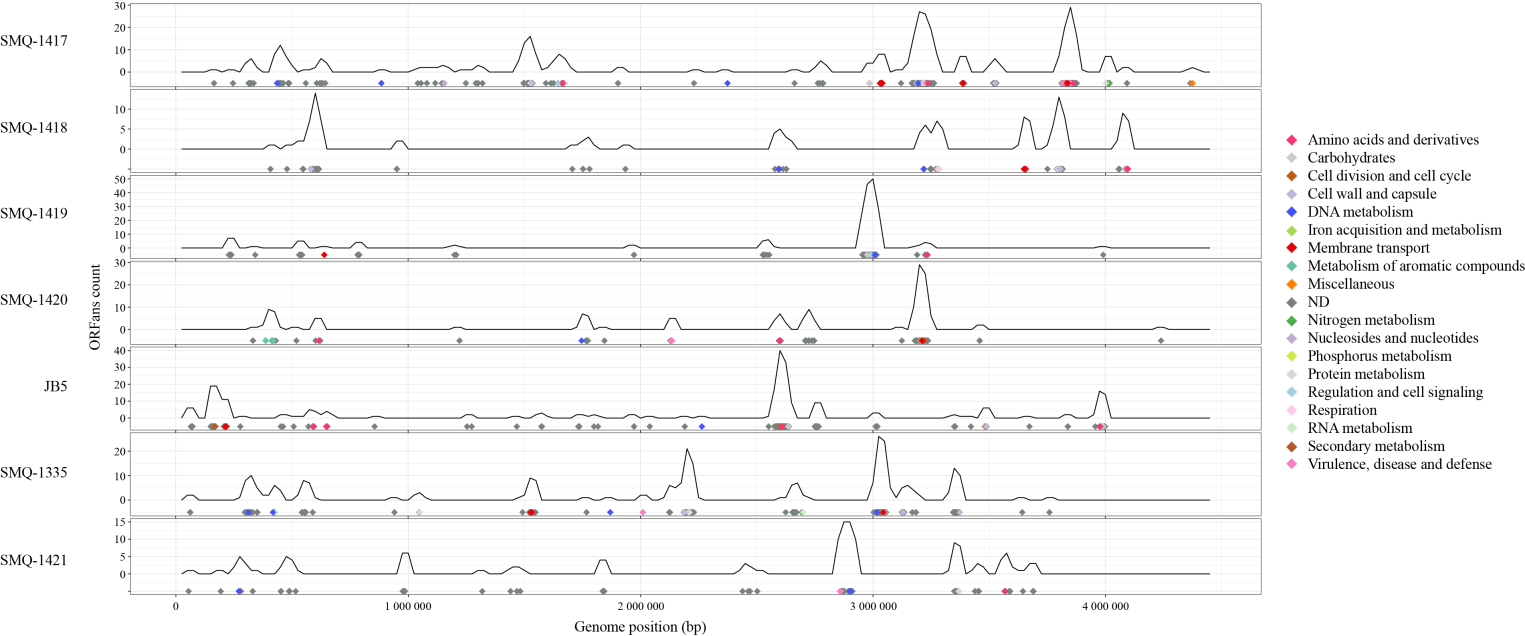
**

**Supplementary Figure S1** **ORFans distribution in *B. aurantiacum* genomes.** ORFans genomic positions and abundance are presented. Diamond colors correspond to RAST subsystem categories.
