# Supplementary material for: Mobilome of Brevibacterium aurantiacum Sheds Light on Its Genetic Diversity and Its Adaptation to Smear-Ripened Cheeses
Source: Front Microbiol. 2019 Jun 10;10:1270. doi: 10.3389/fmicb.2019.01270 (PMC6579920; doi:10.3389/fmicb.2019.01270)
Supplement: Supplementary file 6 [file Data_Sheet_2.docx]

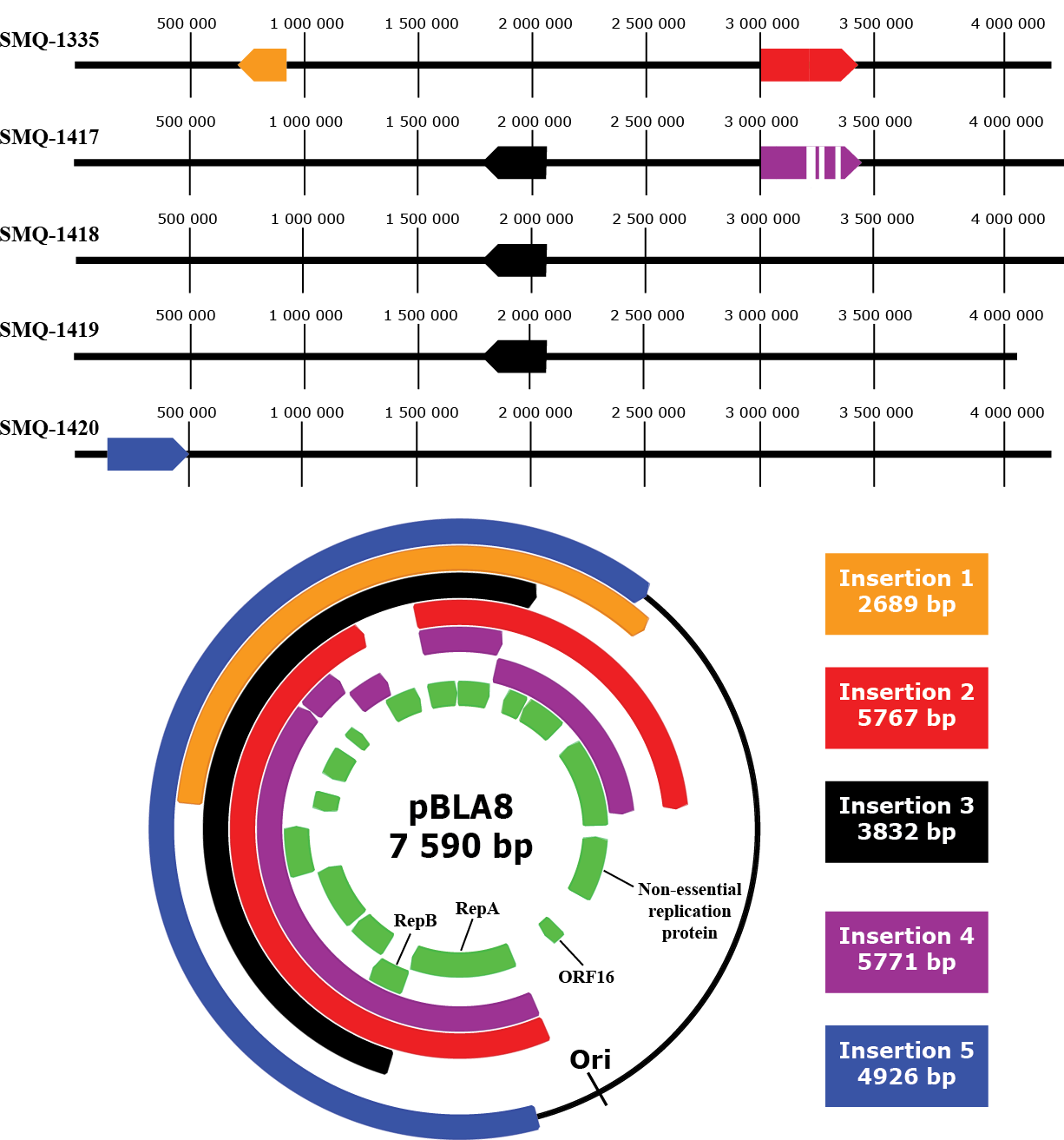


**Supplementary Figure S2** Distribution of pBLA8 fragments in the genomes of *B. aurantiacum* strains. pBLA8 CDS genomic positions and predicted functions are available in Supplementary Table S3.
